# Screening of novel narrow-spectrum benzofuroxan derivatives for the treatment of multidrug-resistant tuberculosis through *in silico*, *in vitro*, and *in vivo* approaches

**DOI:** 10.3389/fmicb.2024.1487829

**Published:** 2024-10-11

**Authors:** Débora Leite Campos, Christian Shleider Carnero Canales, Fernanda Manaia Demarqui, Guilherme F. S. Fernandes, Camila Gonçalves dos Santos, João Lucas B. Prates, Ingrid Gracielle Martins da Silva, Karine Brenda Barros-Cordeiro, Sônia Nair Báo, Leonardo Neves de Andrade, Nathália Abichabki, Luísa Vieira Zacharias, Marli Matiko Anraku de Campos, Jean Leandro dos Santos, Fernando Rogério Pavan

**Affiliations:** ^1^Tuberculosis Research Laboratory, School of Pharmaceutical Sciences, São Paulo State University – UNESP, São Paulo, Brazil; ^2^School of Pharmacy, Biochemistry and Biotechnology, Santa Maria Catholic University, Arequipa, Peru; ^3^Medicinal Chemistry Laboratory, School of Pharmaceutical Sciences, São Paulo State University – UNESP, São Paulo, Brazil; ^4^School of Pharmacy, University College London, London, United Kingdom; ^5^Microscopy and Microanalysis Laboratory, Cell Biology Department, Institute of Biological Sciences, University of Brasilia, Brasília, Brazil; ^6^University of São Paulo – USPSchool of Pharmaceutical Sciences of Ribeirão Preto, , São Paulo, Brazil; ^7^Mycobacteriology Laboratory, Department of Clinical and Toxicological Analysis, Federal University of Santa Maria, Santa Maria, Brazil

**Keywords:** benzofuroxan, multidrug-resistant, tuberculosis, Rv1855c, *Mycobacterium tuberculosis*, narrow-spectrum

## Abstract

Tuberculosis remains a serious global health threat, exacerbated by the rise of resistant strains. This study investigates the potential of two benzofuroxan (*Bfx*) derivatives, 5n and 5b, as targeted treatments for MDR-TB using *in silico*, *in vitro*, and *in vivo* methodologies. *In vitro* analyses showed that *Bfx* compounds have significant activity against *Mtb* H37Rv, with *Bfx* 5n standing out with a MIC_90_ of 0.09 ± 0.04 μM. Additionally, their efficacy against MDR and pre-XDR strains was superior compared to commercial drugs. These *Bfx* compounds have a narrow spectrum for mycobacteria, which helps avoid dysbiosis of the gut microbiota, and they also exhibit high selectivity and low toxicity. Synergism studies indicate that *Bfx* derivatives could be combined with rifampicin to enhance treatment efficacy and reduce its duration. Scanning electron microscopy revealed severe damage to the morphology of *Mtb* following treatment with *Bfx* 5n, showing significant distortions in the bacillary structures. Whole-genome sequencing of the 5n-resistant isolate suggests resistance mechanisms mediated by the Rv1855c gene, supported by *in silico* studies. *In vivo* studies showed that the 5n compound reduced the pulmonary load by 3.0 log_10_ CFU/mL, demonstrating superiority over rifampicin, which achieved a reduction of 1.23 log_10_ CFU/mL. In conclusion, *Bfx* derivatives, especially 5n, effectively address resistant infections caused by *Mtb*, suggesting they could be a solid foundation for future therapeutic developments against MDR-TB.

## Introduction

Tuberculosis (TB), predominantly caused by *Mycobacterium tuberculosis* (*Mtb*), remains one of the leading infectious diseases worldwide (Scarim and Pavan, 2022; Scarim and Pavan, 2022). The global health landscape was significantly impacted by the emergence of SARS-CoV-2, which not only caused a pandemic of its own but also led to a sudden increase in the incidence of many other communicable and non-communicable diseases (Hogan et al., 2020; Singh et al., 2024). One of the diseases that was profoundly affected is TB, due to multiple factors, particularly the interruption of laboratory services, shortages in drug supplies, and the diversion of funding and healthcare personnel toward the diagnosis and care of patients with SARS-CoV-2 (Kaufmann, 2023).

As a consequence of these disruptions, in 2022 approximately 10.6 million people contracted TB, an increase compared to the 10.3 million reported in 2021 and 10 million in 2020. This represents a reversal in the downward trend in cases observed up until 2020. Moreover, between 2020 and 2022, the TB incidence rate increased by 3.9%, which starkly contrasts with the annual decrease of approximately 2% recorded in the previous decade. This change marks a turning point in the international fight against TB (World Health Organization, 2023; Silva et al., 2023; Canales et al., 2024). Additionally, it is estimated that multidrug-resistant TB (MDR-TB) accounts for 13% of all deaths related to antimicrobial resistance globally. This situation arises from both the continuous acquisition of resistance and the transmission of the disease from person to person (Farhat et al., 2024).

The standard treatment for TB includes a combination of antimycobacterial drugs (Shleider Carnero Canales et al., 2023), with a duration ranging from 4 to 24 months, depending on resistance, patient condition, and treatment progress.(Guidelines Review Committee, Global Tuberculosis Programme, 2022). Multidrug therapy is essential due to its ability to rapidly reduce the mycobacterial load, thereby limiting disease transmission. Additionally, this therapy targets persistent mycobacterial populations to minimize the risk of drug resistance (Lee et al., 2020). However, these drugs are associated with numerous adverse effects that can cause severe morbidities and, in extreme cases, even death (Lan et al., 2020). Hepatotoxicity is one of the most common adverse effects, with medications like isoniazid and rifampicin (RFP) known to cause significant liver damage, sometimes leading to severe complications (Zhuang et al., 2022). Additionally, second-line drugs such as amikacin and kanamycin pose risks of renal toxicity and ototoxicity, which can result in kidney damage and hearing los (Rybak et al., 2021; Le et al., 2023). Linezolid, a drug used for treating MDR-TB, has been associated with hematological and neurological toxicities, including anemia and peripheral neuropathy (Carnero Canales et al., 2024).

A particularly concerning and often underestimated side effect in TB treatment is the dysbiosis of the gut microbiota. The balance of the gut microbiota plays a crucial role in the development and regulation of the immune system, including the modulation of innate and adaptive immune responses, T cell differentiation, migration and apoptosis of immune cells, activation of Toll-like receptor signaling, and suppression of inflammatory tone (Comberiati et al., 2021; Liu et al., 2021). Additionally, it is actively involved in regulating energy metabolism and synthesizing essential vitamins (Wang et al., 2020). Dysbiosis not only compromises these vital functions but also increases susceptibility to opportunistic infections, exacerbates chronic inflammation, and contributes to various metabolic disorders, such as obesity and type 2 diabetes (Andremont et al., 2021). This is particularly concerning in TB patients, who are already immunocompromised and vulnerable to the adverse effects of prolonged and aggressive treatment with multiple antimicrobial drugs.

Recent studies have shown that antituberculous drugs cause prolonged and significant damage to the gut microbial community, which can persist for several months after the cessation of treatment, potentially leading to impaired immune response, reinfections, and drug resistance (Diallo et al., 2021; Namasivayam et al., 2017). Given these challenges, an urgent need exists to develop new antimicrobial compounds that are not only effective against resistant strains of *Mtb* but also possess a narrow spectrum of activity to minimize the impact on the gut microbiota (Carlson and Gonzales-Luna, 2020).

In this context, *Bfx* derivatives have emerged as promising candidates. These compounds are heterocyclic structures characterized by the fusion of a benzene ring with a furoxan ring, and they have garnered attention due to their broad range of biological activities, including antimicrobial, antiviral, and anticancer properties (Fernandes et al., 2021; dos Santos Fernandes et al., 2017; Chugunova et al., 2020). Notably, some *Bfx* derivatives have shown potent inhibitory activity against both standard and drug-resistant clinical strains of *Mtb* (Fernandes et al., 2023). The proposed mechanism of action for *Bfx* derivatives involves multiple targets within the mycobacterial cell. One suggested mechanism is the generation of reactive nitrogen species through the release of nitric oxide, leading to nitrosative stress and damage to essential cellular components of *Mtb* (Chugunova et al., 2023).

**Figure 1:**
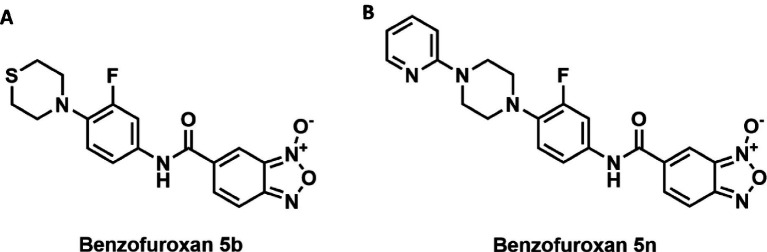
(A) Compound 5b consists of a 2,3,5,6-tetrahydro-1,4-thiazine ring attached to a fluorobenzene ring. This fluorobenzene ring is connected to a benzofuroxan core through an amide bond. (B) Compound 5n is composed of a pyridine ring linked to a piperazine ring. The piperazine, in turn, is attached to a fluorobenzene ring. This fluorobenzene ring is connected to a benzofuroxan core through an amide bond, similar to compound 5b.

This study explores the activities of two specific *Bfx* compounds, 5n and 5b (Figure 1), against *Mtb* in various contexts. *In vitro* analyses targeted drug-resistant clinical strains and intracellular mycobacteria, evaluating the spectrum of action against diverse mycobacterial species and other bacteria. *In vivo* activities were examined using a murine model, and whole-genome sequencing was employed to unravel the genotypic alterations in a 5n-resistant variant. Additionally, *in silico* analyses provided insights into the molecular interactions between *Bfx* 5n and the Rv1855c protein. These *Bfx* derivatives present a promising avenue for advancing treatment modalities and mitigating the global burden of this deadly infection.

## Materials and methods

### Benzofuroxan compounds

The *Bfx* derivatives (5b and 5n) were synthesized by Medical Chemistry Laboratory of Pharmaceutical Sciences of Araraquara (Fernandes et al., 2021). These compounds were provided in their pure form and did not require any additional synthesis in our laboratory.

### Resazurin microdilution assay

The minimum inhibitory concentration (MIC_90_) of the *Bfx* derivatives was evaluated against *Mycobacterium bovis*, *M. smegmatis*, monoresistant, multidrug resistant (MDR), pre-extensively drug-resistant (pre-XDR), and susceptible (H37Rv) strains of *Mtb* following the methodology of Palomino et al. (2002) with some modifications detailed below. The compounds were serially diluted, with concentrations ranging from 0.09 to 25 μg/mL, in 96-well plates using Middlebrook 7H9 broth supplemented with 10% Oleic acid-albumin-dextrose-catalase (OADC). A suspension of the bacterial strains was added to each well, and the plates were incubated for 7 days (for H37Rv, clinical isolates, and *M. bovis*) or 2 days (for *M. smegmatis*) at 37°C in a 5% CO_2_ atmosphere. Subsequently, resazurin (0.01% in sterile water) was added. Resazurin is a redox indicator that is reduced to resorufin by metabolically active bacteria, resulting in a measurable fluorescence signal that correlates with cell viability. After 24 h (for H37Rv, clinical isolates, and *M. bovis*) or 12 h (for *M. smegmatis*), fluorescence was measured at 530/590 nm using a Cytation 3 microplate reader (Biotek®).

### Antimicrobial evaluation against gram-positive, gram-negative bacteria, and *Candida albicans*

The methodology employed in this study was based on the protocols established by Ben Hur et al. (2022), using Mueller-Hinton II broth for most of the bacteria studied. For more demanding bacteria, the medium was supplemented with lysed horse blood and *β*-NAD (MH-F broth) (Carkaci et al., 2017). Detailed descriptions of the methodologies and specific conditions used in the experiments can be found in the Supplementary material.

### Cytotoxicity assay and determination of selective index

Murine macrophage J774.A1 and MRC-5 fibroblasts cells were seeded in 96-well plates (1.0 × 10^6^ cells/mL) and incubated for 24 h at 37°C, 5% CO₂ for adherence. Compounds were applied to the wells at concentrations ranging from 0.39 to 100 μg/mL. After 24 and 72 h, 30 μL of resazurin was added. Fluorescence was measured after 4 h at 530/590 nm using a Cytation 3 (Biotek®) reader. The IC_50_ was determined as the minimum compound concentration capable of inhibiting 50% of cell viability compared to the control. The SI was calculated as IC_50_/MIC_90_, considering compounds with SI > 10 as safe (Amaral et al., 2024).

### Checkerboard assay

To evaluate the interaction between Bfx and RFP, a checkerboard assay was conducted following the methodology proposed by Berenbaum (Berenbaum, 1978).Test compounds were serially diluted in Middlebrook 7H9 broth and arranged in a 96-well plate, starting with 2x MIC for each compound. RFP was also diluted horizontally based on its MIC_90_. The combinations of different concentrations of *Bfx* and RFP were incubated with *Mtb* (5 × 10⁵ CFU/mL) at 37°C and 5% CO₂ for 7 days. Post-incubation, 0.01% resazurin was added, and fluorescence readings were taken after 24 h to determine the Fractional Inhibitory Concentration Index (FICI). The combined effects of the compound combinations were classified according to the Fractional Inhibitory Concentration Index (FICI):


$$\mathrm{FICI}=\frac{\begin{array}{l} \mathrm{MIC}\;\mathrm{of}\;\mathrm{drug}\;1\;\mathrm{in}\;\\ \mathrm{combination} \end{array}}{\mathrm{MIC}\;\mathrm{of}\;\mathrm{drug}\;1}+\frac{\begin{array}{l} \mathrm{MIC}\;\mathrm{of}\;\mathrm{drug}\;2\;\mathrm{in}\;\\ \mathrm{combination} \end{array}}{\mathrm{MIC}\;\mathrm{of}\;\mathrm{drug}\;1}$$

### Intracellular antimycobacterial activity

Murine macrophage J774.A1 cells (1 × 10^6^ cells/mL) were seeded in 24-well plates and incubated as previously described. After 24 h, the supernatant was discarded, and 1 mL of *Mtb* inoculum (2 × 10^6^ CFU/mL) was added to the wells. The plate was incubated for 2 h to facilitate infection. Subsequently, the supernatant was removed, and the cells were washed with PBS. An amikacin solution (200 μg/mL) was added to ensure the sterility of the extracellular environment. After 2 h, the wells were washed, and treatments were applied (1x, 5x, and 10x MIC_90_ of *Bfx* or RFP). The cultures were then incubated for 72 h. After incubation, the cells were lysed using a Triton solution, and the supernatant was collected, serially diluted, and plated on Middlebrook 7H11 medium supplemented with 10% OADC. These plates were incubated for 20–30 days until colony-forming units (CFU) were counted (Solcia et al., 2021).

### Cell membrane evaluation by scanning electron microscopy

*Mtb* cultures (5 mL at 10⁵ CFU/mL) were prepared and divided into four groups: one treated with *Bfx*, one with isoniazid (INH), another with ethambutol (EMB), and an untreated group, following the described conditions. After incubation, the cells were washed with PBS and fixed overnight with Karnovsky fixative solution (containing 2% glutaraldehyde, 2% paraformaldehyde, and 3% sucrose in 0.1 M sodium cacodylate buffer, pH 7.2). The cells were then washed with 0.1 M sodium cacodylate buffer, pH 7.2, postfixed with 2% osmium tetroxide for 30 min, and washed with distilled water. Dehydration was carried out in an increasing series of acetone (50–100%), followed by critical point drying using CPD 030 (BALZERS, Geneva, IL, United States) and SCD 500 metallization (LEICA, Wetzlar, Hesse, Germany), before being analyzed using a JSM 7001F scanning electron microscope (15 kV) (Jeol—Tokyo, Japan) (Caleffi-Ferracioli et al., 2016).

### Isolation of Bfx-resistant Mtb and whole genome sequencing

To select *Mtb* strains resistant to the most active *Bfx* compounds, a bacterial inoculum at 10⁵ CFU/mL was seeded on Middlebrook 7H11 agar plates supplemented with OADC and various concentrations of compound 5n (0.78 to 25 μg/mL). Plates were incubated at 37°C in a 5% CO₂ atmosphere until bacterial growth was observed. A colony that grew at concentrations above the MIC_90_, as determined by the REMA method, was identified as a resistant mutant. DNA was then extracted from the resistant colony using glass beads. The colony was vortexed with TE buffer and beads, followed by supernatant transfer, precipitation with sodium acetate and ethanol, and incubation at −20°C. After centrifugation, the supernatant was discarded, and the pellet was washed with 70% ethanol, dried, and resuspended in milliQ water. The DNA concentration and quality were assessed using a Nanodrop spectrophotometer and stored at −20°C. Whole Genome Sequencing was conducted at SeqCenter (Pittsburgh, PA, United States) using Illumina HiSeq technology with a coverage depth greater than 100. Variant calling was performed with Breseq software, comparing the sequences to the reference *Mtb* H37Rv genome (Deatherage and Barrick, 2014).

### *In silico* analysis

The 2D structure of compound 5n was designed using ChemDraw Professional 17.0, and its 3D analog was generated in Chem3D (Cousins, 2005). The molecular geometry was energy optimized using the auto-optimization tool in Avogadro software, applying a universal force field (Hanwell et al., 2012). The modeling of the Rv1855c protein was performed using the I-TASSER web server (Yang and Zhang, 2015).^1^ To determine the druggability of the Rv1855c protein pockets and their binding affinity with compound 5n, PockDrug was used (Hussein et al., 2015),^2^ selecting pockets with a probability greater than 95% and a minimum proximity of 5.5 Å (Primo et al., 2023). Molecular docking analysis was conducted with AutoDock Vina (Trott and Olson, 2010; Eberhardt et al., 2021). Finally, ligand-receptor interactions were analyzed and visualized using Discovery Studio Visualizer software (Pawar and Rohane, 2021).

### *In vivo* infection and treatment

Female BALB/c mice (7–8 weeks old) were housed under controlled conditions and stratified into groups: Infection Control (IC), Post-Infection Control (PIC), RFP, 5b, and 5n, each in triplicate. Mice were infected intranasally with 20 μL of Mtb H37Rv suspension (1.5 × 10⁵ CFU/mouse). Fourteen days post-infection, the IC group was sacrificed to measure the bacterial load present in their lungs at the beginning of the experimental treatment. Treatment groups received daily oral suspensions of 5b or 5n at 200 mg/kg/mouse, and RFP at 15 mg/kg/mouse. Control groups received saline solution. The PIC group was sacrificed on day 42 to compare how the infection evolves over time without active treatment. After completing the experimental timeline, lung tissues were collected, homogenized, and cultured on Middlebrook 7H11 agar to determine the bacterial load, with incubation at 37°C and 5% CO₂ for up to 60 days to count CFU (Mourik et al., 2018).

### Statistics and reproducibility

The results presented here are expressed as the mean ± standard deviation (SD) of three experimental analyses. Statistical significance analyses were performed using GraphPad Prism 5.01 (GraphPad Software Inc., La Jolla, CA) through a one-way Analysis of Variance (ANOVA) followed by Dunnett’s *post hoc* test. Statistical significance was considered for *p*-values less than 0.05.

## Results

### Bfx compounds exhibit powerful activity against resistant clinical isolates of Mtb

The *Bfx* compounds demonstrated significant potential in inhibiting monoresistant strains to INH, MDR, and Pre-XDR, as well as the drug-sensitive *Mtb* strain H37Rv, as shown in Table 1. Compound 5n exhibited high efficacy against most clinical isolates, except for the MDR strain CF48, where it reached an inhibitory concentration of 22.38 ± 9.37 μM. However, it stood out for its ability to eliminate Pre-XDR strains at concentrations as low as 0.28 μM, outperforming all the commercial drugs tested, including RFP and LNZ, which showed significantly higher values.

**Table 1:** CIM_90_ values (μM) of *Bfx* compounds and commercial drugs against sensitive mycobacteria and clinical isolates of *Mtb.*

Class.	Mono INH	Mono INH	MDR	MDR	MDR	MDR	MDR	MDR	Pre-XDR	Pre-XDR	Sensitive	Sensitive	Sensitive
Comp.	*CF* 44	*CF* 184	*CF* 61	*CF* 48	*CF* 165	*CF* 171	*CF* 162	*CF* 105	*CF* 169	*CF* 81	*Mtb* H37Rv	*M. smegmatis*	*M. bovis*
5n	4,71 ± 3,11	0,96 ± 1,04	0,56 ± 0,47	22,38 ± 9,37	1,84 ± 1,73	0,26 ± 0,05	2,24 ± 1,47	> 57,26	0,28 ± 0,10	1,68 ± 1,32	0,09 ± 0,04	41,07 ± 10,05	27,03 ± 10,43
5b	0,52 ± 0,09	0,37 ± 0,15	0,28 ± 0,02	22,76 ± 13,20	0,24 ± 0,00	0,25 ± 0,01	0,26 ± 0,00	> 66,78	0,40 ± 0,03	0,27 ± 0,00	0,70 ± 0,26	24,17 ± 13,00	0,85 ± 0,61
LNZ	ND	ND	ND	33,88 ± 1,27	0,95 ± 0,01	0,58 ± 0,02	0,41 ± 0,03	4,15 ± 0,52	0,95 ± 0,59	0,47 ± 0,32	0,5 ± 0,20	1,0 ± 0,20	0,25 ± 0,1
RFP	0,05 ± 0,01	0,05 ± 0,01	> 30,38	> 30,38	> 30,38	> 30,38	> 30,38	> 30,38	6,95 ± 0,19	> 30,38	0,05 ± 0,01	1,21 ± 0,00	0,10 ± 0,01
INH	> 182,30	> 182,30	> 182,30	> 182,30	71,56 ± 24,71	> 182,30	> 182,30	> 182,30	> 182,30	> 182,30	0,95 ± 0,43	2,0 ± 0,50	0.2 ± 0,01
MO/GA	0,24 ± 0,00	0,61 ± 0,07	3,37 ± 0,69	3,11 ± 0,36	0,40 ± 0,04	2,48 ± 1,12	4,69 ± 4,17	0,25 ± 0,01	10,64 ± 4,96	41,60 ± 35,81	0,5 ± 0,25	0,75 ± 0,25	0,4 ± 0,20
AMK	1,48 ± 1,12	0,24 ± 0,00	> 42,69	12,27 ± 6,77	5,33 ± 0,95	0,24 ± 0,10	> 42,69	7,54 ± 11,48	0,36 ± 0,11	19,52 ± 12,55	3,0 ± 1,00	6,0 ± 2,00	1,5 ± 0,50

*Class, classification; Comp, compound; ND, not determined; LNZ, linezolid; RFP, rifampicin; INH, isoniazid; MO, moxifloxacin; GA, gatifloxacin; AMK, amikacin. Mono INH, strains classified as mono-resistant to isoniazid; MDR, multidrug-resistant strains; XDR, extensively drug-resistant strains.

On the other hand, compound 5b not only demonstrated potent activity against *Mtb* H37Rv (0.70 ± 0.26 μM) but was also effective against multiple MDR and Pre-XDR strains, such as CF165 (0.24 ± 0.00 μM), CF171 (0.25 ± 0.01 μM), and CF162 (0.26 ± 0.00 μM). These low inhibitory concentrations suggest that the structural modifications in compound 5b may enhance its ability to evade the specific resistance mechanisms presented by these strains. It is important to note that strains CF165 and CF171 are resistant to INH and RFP; CF61 and CF162 are resistant to amikacin; CF48 and CF105 are resistant to both amikacin and linezolid; and CF81 and CF169 are resistant to RFP, INH, and a fluoroquinolone.

### The Bfx compounds exhibit narrow-spectrum antimicrobial activity against mycobacteria

Compounds 5n and 5b were evaluated against a variety of Gram-negative, Gram-positive bacteria, and fungi, including *Candida albicans*. According to the data presented in Table 2, both compounds showed a lack of significant activity against all the bacterial and fungal strains tested, with MIC_90_ values exceeding 200 μM for all species analyzed. In contrast, commercial drugs such as RFP exhibited notable activities, particularly against *Staphylococcus epidermidis* (0.001 μM) and *Staphylococcus saprophyticus* (0.009 μM).

**Table 2:** MIC_90_ values of *Bfx* compounds against *Candida albicans*, gram-negative and gram-positive bacteria.

Strains	5n (μM)	5b (μM)	RFP (μM)	INH (μM)
*Klebsiella pneumoniae subsp. pneumoniae ATCC 13883 T*	> 200	> 200	5,1	> 500
*Klebsiella oxytoca CCT 0182*	> 200	> 200	7,5	> 500
*Staphylococcus saprophyticus subsp. saprophyticus ATCC 15305*	> 200	> 200	0,009	> 500
*Staphylococcus epidermidis ATCC 14990 T*	> 200	> 200	0,001	> 500
*Enterobacter cloacae subsp. cloacae ATCC 13883 T*	> 200	> 200	> 100	> 500
*Enterobacter aerogenes ATCC 13048 T*	> 200	> 200	> 100,0	> 500
*Enterococcus faecium NCTC 7171 T*	> 200	> 200	> 100,0	> 500
*Enterococcus faecalis ATCC 29212*	> 200	> 200	0,004	> 500
*Proteus mirabilis ATCC 29906 T*	> 200	> 200	19,4	> 500
*Citrobacter freundii ATCC 8030 T*	> 200	> 200	38,9	> 500
*Candida albicans ATCC 90028*	> 200	> 200	ND	ND

*ND, not determined.

These results indicate that compounds 5n and 5b do not have a broad spectrum of action. On the contrary, when comparing these data with the previous results obtained against *Mtb*, where both compounds demonstrated high efficacy, it can be inferred that 5n and 5b might act as narrow-spectrum antimicrobial agents. This suggests that the mechanisms of action of 5n and 5b may be highly specialized for *Mtb*, avoiding disruption of the gut microbiota and potentially reducing adverse effects associated with broad-spectrum treatments. This narrow focus could also minimize the risk of cross-resistance with other classes of antibiotics, a key factor in combating the growing threat of antimicrobial resistance.

### Bfx exhibits synergistic interaction with RFP against Mtb and demonstrates a high selectivity index

Compound 5n showed an IC_50_ greater than 230.24 μM in murine macrophages J774.A1 and 221.52 μM in human fibroblasts MRC-5. These values indicate a low cytotoxicity of the compound toward healthy cells. The selectivity indices (SI) greater than 2,400 for both cell lines reflect a remarkable ability of the compound to inhibit *Mtb* H37Rv (MIC_90_ of 0.09 ± 0.04 μM) without significantly affecting healthy cells (Table 3). This indicates that compound 5n has a wide therapeutic margin.

**Table 3:** Profile of antimicrobial activity, cytotoxicity, and selectivity of *Bfx* against *Mtb* in MRC-5 and J774.A1 cell lines.

Compound	MIC_90_ *Mtb* H37RV	IC_50_ J774.A1	IC_50_ MRC-5	SI J774.A1	SI MRC-5
5n	0,09 ± 0,04	> 230,24	> 221,52	>2,400	>2,400
5b	0,7 ± 0,26	40,83 ± 10,72	57,07 ± 48,15	57.14285714	81.428571
RFP	0,05 ± 0,01	> 121,51	> 121,51	>2,400	>2,400
INH	0,95 ± 0,44	> 729,18	> 729,18	>750	>750

RFP, rifampicin; INH, isoniazid. Values expressed in μM.

Compound 5b, on the other hand, presented an IC_50_ of 40.83 ± 10.72 μM in J774.A1 and 57.07 ± 48.15 μM in MRC-5. Although these values reflect higher cytotoxicity compared to 5n, the calculated selectivity indices of 57.14 for J774.A1 and 81.43 for MRC-5 still indicate an acceptable therapeutic margin. The antimicrobial activity of compound 5b, with an MIC_90_ of 0.7 ± 0.26 μM against *Mtb* H37Rv, suggests that despite the relatively higher toxicity, it remains a viable compound for the study in the inhibition of *Mtb*, provided that doses are carefully considered to minimize adverse effects.

To explore the combined effectiveness of these compounds with RFP, a checkerboard assay was conducted to evaluate the synergy between *Bfx* and RFP against *Mtb* H37Rv. The results showed FICI values of 0.083 for compound 5n and 0.11 for compound 5b, indicating significant synergy (Supplementary Table S1). This suggests that the combination of *Bfx* with RFP could enhance treatment efficacy, allowing for the use of lower concentrations of both compounds, potentially contributing to a reduction in overall treatment toxicity.

### Treatment with 5n causes severe damage to the morphology of Mtb

**Figure 2:**
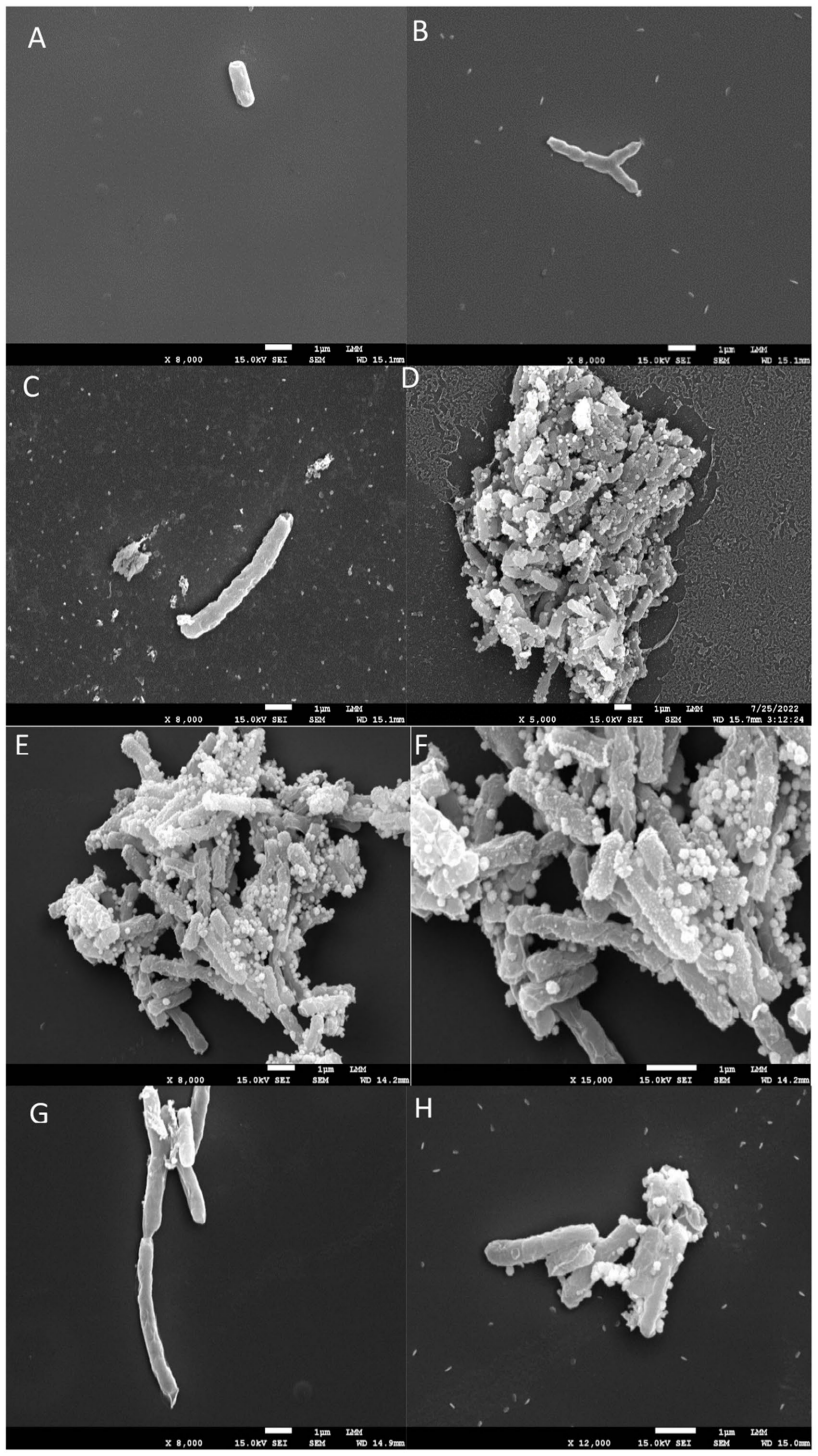
Scanning electron microscopy images. (A,B) shows untreated *Mycobacterium tuberculosis* bacilli, displaying their characteristic rod-shaped morphology. (C–H) illustrates bacilli after 24 h of exposure to INH, EMB, and 5n at MIC_90_ values. Treatment with compound 5n caused morphological damage to *Mtb*, resulting in distorted filamentous structures and compromised bacilli integrity.

*Mtb* was treated with compounds EMB, INH, and 5n, as well as an untreated control, all at concentrations equivalent to 1x MIC (Figure 2). The purpose of these experiments was to evaluate the morphological effects induced by these compounds on *Mtb* using scanning electron microscopy (SEM). The obtained images allowed for a comparison of the structural integrity of untreated mycobacteria with the morphological alterations caused by the treatments, thus providing a clearer view of the efficacy and possible mechanisms of action of each compound.

In the untreated control (Figures 2A,B), the mycobacteria exhibited a typical bacillary morphology, with intact and well-defined cellular structures, without any signs of structural damage. These images served as a reference to assess the effects of the antimicrobial compounds.

Treatment with EMB at 1x MIC (Figures 2C,D) showed damaged cell surfaces and the presence of cellular debris, suggesting a bactericidal effect characterized by disruption of cell wall integrity. The disruption in bacillary morphology, along with the disorganization of the cell surface, indicates that EMB exerts its action through interference with the synthesis of the *Mtb* cell wall, resulting in the loss of bacterial viability.

Treatment with INH at 1x MIC (Figures 2E,F) resulted in the appearance of amorphous structures and cell lysis. The treated mycobacteria showed a complete loss of morphology and the formation of disintegrated cellular debris, consistent with a potent lytic effect. This result supports the known mechanism of action of INH, which inhibits the synthesis of mycolic acids essential for the structure and function of the *Mtb* cell wall.

Finally, treatment with compound 5n at 1x MIC (Figures 2G,H) also caused morphological damage to *Mtb*. Mycobacteria treated with 5n showed distorted filamentous structures and considerable damage to the integrity of the bacilli. These observations suggest that 5n induces a unique mechanism of action that may involve the disruption of critical components of the *Mtb* cell wall or plasma membrane, resulting in deformation and possible cell death.

### Genomic analysis unveils mechanistic insights into resistance in an Mtb isolate against Bfx 5n

To evaluate the mechanism of action of 5n, we aimed to isolate a *Bfx*-resistant mutant. 7H11 agar plates with varying concentrations of the compound were prepared, and we successfully isolated a colony capable of growing at a concentration of 13.5 μM. This colony appeared on the solid medium 40 days after inoculation, was collected, and re-inoculated onto a plate containing 13.5 μM of compound 5n. New colonies were obtained; some were used for genetic material extraction for sequencing, while others were cultured for storage. We considered these colonies to be 5n-resistant mutants due to their growth on a medium containing more than 150 times the previously determined MIC of 0.09 μM. The genetic material was subjected to whole-genome sequencing and variant analysis using Breseq (Deatherage and Barrick, 2014). Comparative analysis was conducted between (a) the reference genome of *Mtb* H37Rv available at https://www.ncbi.nlm.nih.gov/datasets/genome/GCF_000195955.2/ (reference strain); (b) the *Mtb* H37Rv genome from our laboratory used as a control (control strain); and (c) the genome of the 5n-resistant mutant obtained in the previous procedure (5n Mutant). This analysis revealed four single nucleotide polymorphisms (SNPs) among the mutated genes in the control strain and the 5n Mutant.

In the control strain, mutations were identified in the PPE protein family genes (PPE13 and PPE18 at two distinct positions) and in Rv1855c. It is important to note that the genetic alterations identified in these genes did not induce phenotypic changes in their final protein products, as the SNPs were classified as silent mutations. These alterations did not impact the final amino acid sequence during protein translation. However, in the Rv1855c gene, a single base pair deletion was observed, resulting in a frameshift mutation in a hypothetical oxidoreductase. This genomic alteration observed in the laboratory control strain H37Rv did not hinder the *in vitro* growth of the inoculum; on the contrary, it allowed for the optimization of mycobacterial growth. We hypothesize that *Bfx* 5n exerted a “restorative function” by reversing the mutation in the control strain, as the strain needed to protect itself and reduce nitrosative stress. However, to understand the 5n-receptor interactions, we decided to perform complementary *in silico* analyses, as the results observed in the genomic sequencing data analysis are not yet sufficient to confirm the true target and mechanism of action of *Bfx* 5n but shed light on potential resistance mechanisms generated by *Mtb* against this compound.

### Bfx 5n exhibits high *in silico* affinity for the Rv1855c protein of Mtb

**Figure 3:**
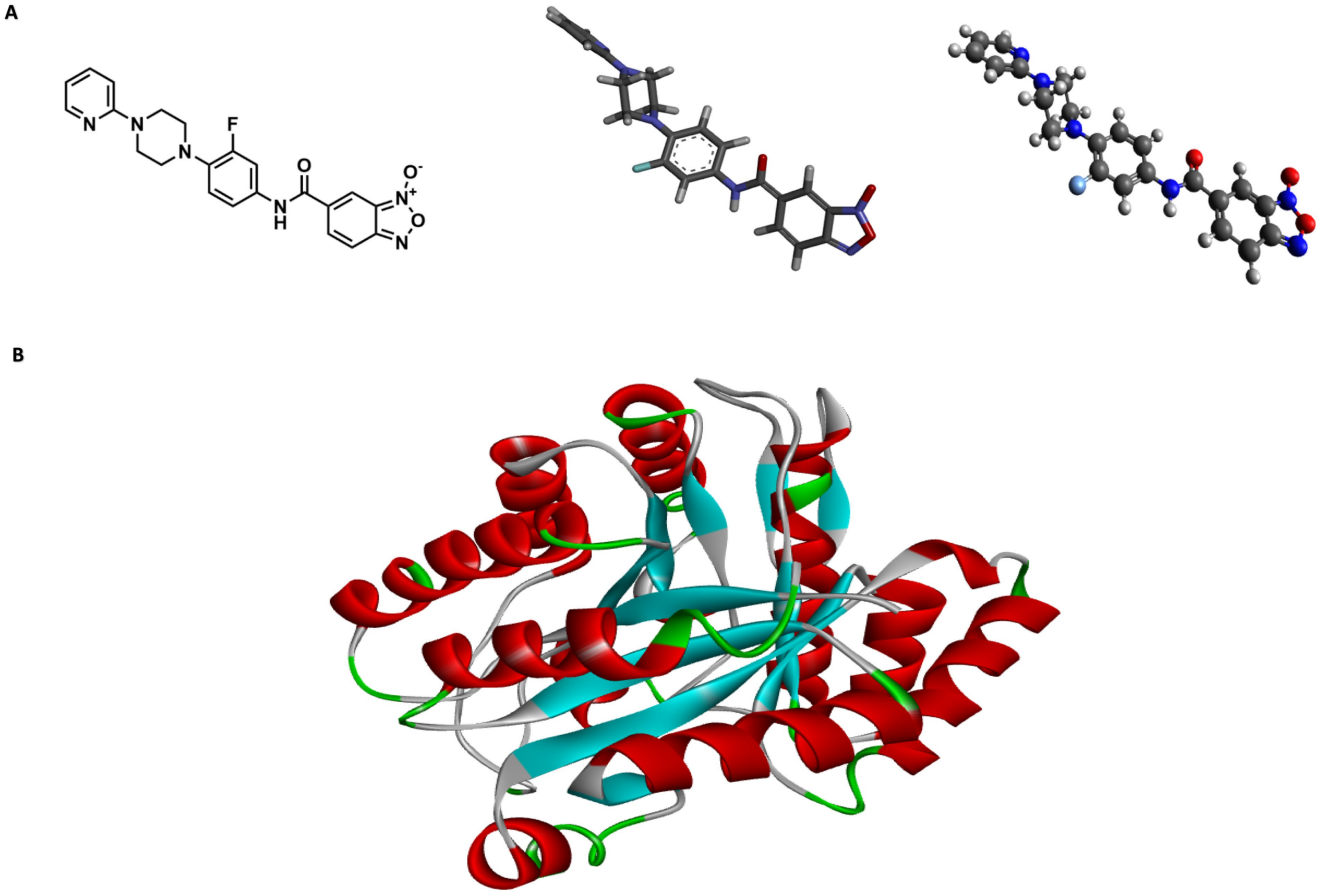

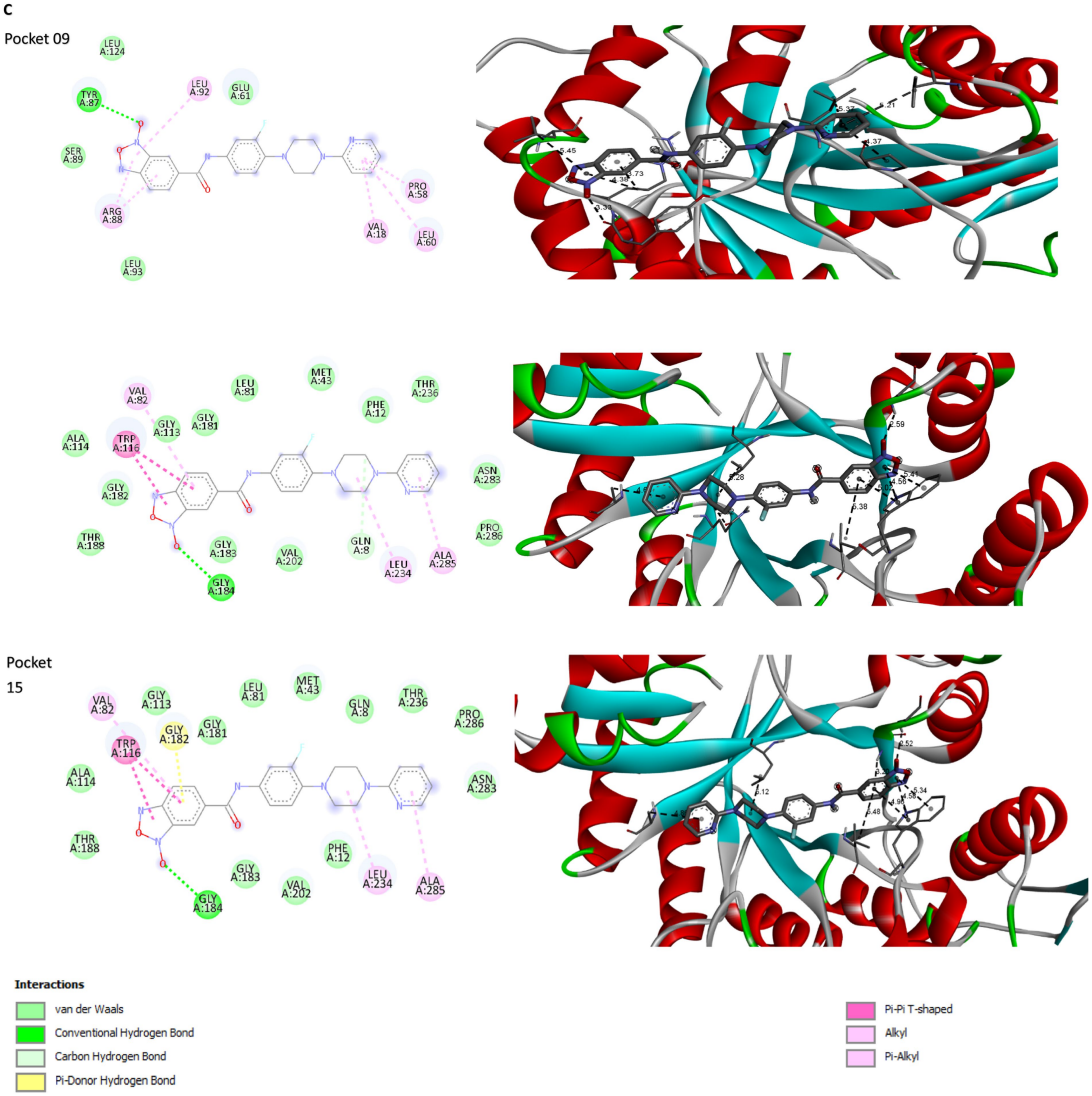
Structural modeling of compound 5n and protein Rv1855c. (A) Depiction of compound 5n showing the transition from its two-dimensional structure to three-dimensional, followed by its optimized form using Avogadro software. (B) Three-dimensional structure of protein Rv1855c, highlighting its complete spatial configuration. (C) 2D and 3D interactions between the ligand and the druggability pockets of receptor. The compound 5n demonstrated an affinity of up to −8.2 kcal/mol for the Rv1855c receptor.

To support the genetic sequencing results and the potential mechanisms of action, *in silico* studies were conducted (Figure 3). The 2D structure of compound 5n was converted to 3D using Chem3D software, and Avogadro was used to relax and optimize the compound (Figure 3A). I-TASSER provided three structural models of the protein encoded by the Rv1855c gene (Rv1855c Protein) with C-scores of 0.69, −3.65, and − 5. Based on the manufacturer’s guidelines, we selected the structure with a C-score of 0.69 (Figure 3B). Distances, classifications, types, and quantities of interactions were visualized using Discovery Studio Visualizer. 2D and 3D images of the ligand-receptor interactions for each of the tested pockets were obtained (Figure 3C).

PockDrug identified 17 pharmacodynamic pockets (Supplementary Table S2), of which only those with a probability greater than 95% of being pharmacodynamic sites were selected. Specifically, pockets 9, 12, and 16 were chosen due to their high pharmacodynamic probabilities of 99, 98, and 95%, respectively. The selection of docking models obtained from AutoDock Vina was determined using three main criteria: ligand affinity for the receptors, the number of simultaneous interactions between the amino acids in each pharmacological pocket and the ligand, and the specific type of bonds formed. AutoDock Vina generated nine models for each molecular docking performed between the ligand and the selected receptor pockets (Supplementary Table S3). 5n bound strongly and stably to pharmacodynamic pockets 9, 12, and 15 of the Rv1855c protein of *Mtb*, with binding energies of −7.6, −8.1, and − 8.2 kcal/mol, respectively. The distances, rankings, types, and quantities of interactions were visualized using Discovery Studio Visualizer. 2D and 3D images of the ligand-receptor interactions were obtained for each of the tested pockets (Figure 3C).

Compound 5n exhibited a variety of interactions with different pockets of the Rv1855c protein (Supplementary Table S4). In pocket 9, seven specific interactions were observed, notably a hydrogen bond with TYR87, characterized by its strength and stability, with a distance of 3.326 Å. The other six interactions in this pocket were of the hydrophobic pi-alkyl type with residues such as ARG88, LEU92, VAL18, PRO58, LEU60, and again ARG88, demonstrating the compound’s tendency to engage in nonpolar interactions. In pocket 12, compound 5n produced eight interactions, including a conventional hydrogen bond with GLY184, with a distance of 2.59085 Å, and a carbon-hydrogen bond with GLN8, with a distance of 3.52464 Å. The remaining six interactions were hydrophobic, highlighting multiple pi-pi T-shaped interactions with the TRP116 residue, suggesting significant interaction with aromatic rings. Finally, in pocket 15, eight interactions were recorded, where compound 5n formed a conventional hydrogen bond with GLY184 and a pi-donor hydrogen bond with GLY182, with distances of 2.52025 Å and 3.19934 Å, respectively. Additionally, six hydrophobic interactions were observed, with pi-pi T-shaped interactions with TRP116 being particularly notable, emphasizing the relevance of the compound’s spatial configuration and its ability to integrate into the hydrophobic environment of the protein pocket.

### Bfx compounds exhibit greater intracellular and *in vivo* activity compared to RFP

**Figure 4:**
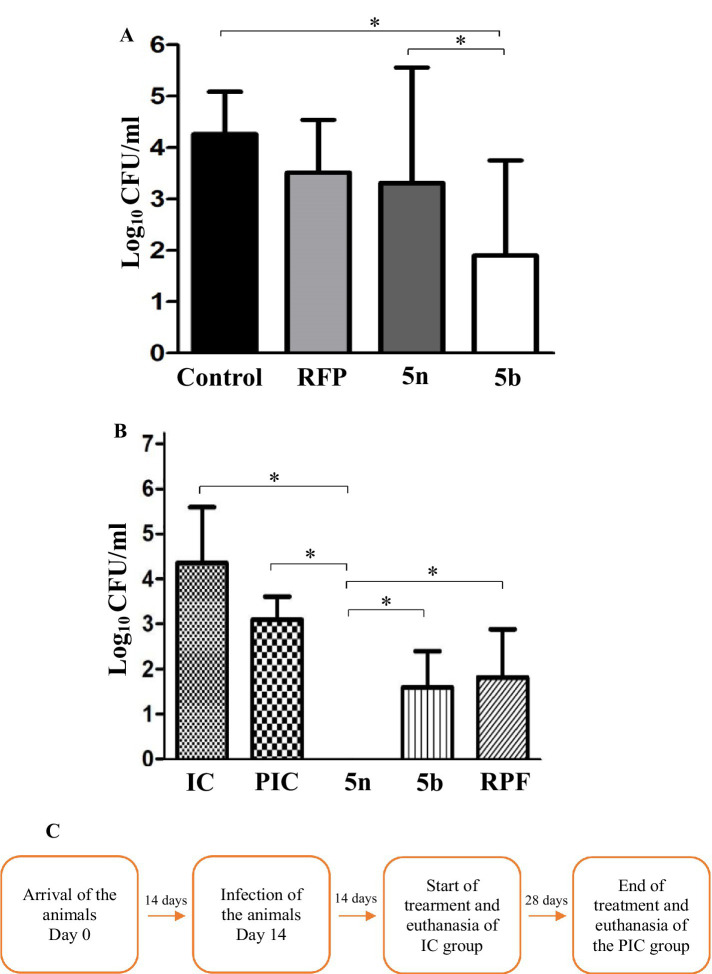
(A) CFU/mL in log_10_ after infection and treatment of J774A.1 macrophages. Non-treated (control) represents 100% bacterial growth within the macrophages. Compounds were evaluated at their minimum inhibitory concentration value at 10x. Mean and standard deviation were obtained from a biological triplicate. (B) CFU after 4 weeks of treatment, obtained by lysing and culturing the lungs of the mice. The groups were divided into IC (14 days post-infection), PIC (42 days post-infection), and treatments with 5n, 5b, and RFP. 5n showed a greater reduction in CFU than 5b and RIF. One-way ANOVA followed by Dunnett’s HSD *post hoc* test was used for statistical analysis in both processes (*p < 0.05). (C) Timeline of the animal study and euthanasia process.

In intramacrophagic studies, the bacterial load within macrophages was evaluated following treatment with RFP at 10 times its MIC, resulting in a 0.7 log_10_ inhibition. Compound 5n showed a 0.9 log_10_ inhibition, while compound 5b achieved a bacterial growth reduction of 2.3 log_10_. These results highlight the efficacy of 5n and 5b, compared to RFP, demonstrated greater potency against intracellular *Mtb* H37Rv (Figure 4A).

In the *in vivo* analyses, Balb/C mice were infected with *Mtb* H37Rv and administered the corresponding treatments, following a protocol detailed in the methodology (Figure 4B). During the four-week treatment, no adverse changes in behavior or animal loss were observed, suggesting good tolerability of the compounds. Initially, 1.5 × 10^5^ CFU per animal were inoculated, and 14 days later, the infected control group (IC) presented a bacterial count in the lungs of 1.4 × 10^5^ CFU (4.2 log_10_). At the end of the 4 weeks, the mean bacterial concentration in the lungs of the mice in the PIC group was 1.5 × 10^3^ CFU (3.0 log_10_), representing a 1.22 log_10_ reduction attributable to the activation of the mice’s immune system, capable of reducing the bacterial load in lung tissue. When comparing the effects of the treatment with the compounds, a 1.23 log_10_ reduction was observed with RFP, a 1.45 log_10_ reduction with 5b, and a 3.0 log_10_ reduction with 5n. This difference in the efficacy of 5n and 5b may be related to their molecular structures, where the presence of a pyridine in 5n could be contributing to its higher bactericidal activity compared to the sulfide group present in 5b (Patel et al., 2020; Marinescu and Popa, 2022; Villamizar-Mogotocoro et al., 2020).

## Discussion

In this study, the antimycobacterial activity of the *Bfx* derivatives 5n and 5b was evaluated, focusing on resistant *Mtb* strains using *in silico*, *in vitro*, and *in vivo* methodologies. These compounds demonstrated promising therapeutic potential. The *in vitro* analyses revealed that *Bfx* derivatives possess potent antimicrobial activity against various *Mtb* strains, including MDR and pre-XDR strains. Compound 5n was particularly notable, with a MIC_90_ of 0.09 ± 0.04 μM, surpassing the efficacy of commercial drugs. Meanwhile, the 5b derivative presented a MIC_90_ of 0.85 ± 0.61 μM against *M. bovis*, which is particularly noteworthy given that this mycobacterium is universally resistant to pyrazinamide (Kanipe and Palmer, 2020). It is important to note that in 2019, approximately 140,000 new cases and 12,500 deaths caused by this pathogen were reported worldwide (Riopel et al., 2024). For *M. smegmatis*, the derivatives exhibited low inhibitory activity (MIC = 24.7–41.07 μM), consistent with Altaf et al. (2010), who reported that these strains are generally less sensitive to *Mtb*-inhibitory compounds. Notably, compound **5n** effectively inhibited the growth of most clinical isolates, including a pre-XDR strain at a MIC of 0.28 ± 0,10 μM. These findings are in line with De Souza et al. (2020), who demonstrated the potential of furoxans against MDR-Mtb strains in murine models. Moreover, the antimycobacterial activity of the benzofuroxans may be enhanced by its ability to modulate NO release, suggesting its potential in designing hybrids that act as both nitric oxide donors and therapeutic agents (Chugunova et al., 2015).

Both compounds demonstrated remarkable specificity, as they did not exhibit antimicrobial activity against Gram-positive or Gram-negative bacteria, nor against *Candida albicans*. This specificity highlights their potential as effective and safer options for the treatment of TB, by reducing the risk of developing resistance in non-target pathogens. According to Melander et al. (2018) narrow-spectrum antibiotics have the advantage of being highly specific for particular types of bacteria, which lowers the likelihood of selecting resistance in non-target organisms and minimizes the negative impact on the host microbiota. In contrast, broad-spectrum antibiotics, while effective against a variety of pathogens, present significant disadvantages. Raymann et al. (2017) report that these drugs can cause prolonged, and even irreversible, disruption of the host microbiota. Additionally, Hotinger et al. (2021) emphasize that the use of broad-spectrum antibiotics increases the likelihood of inducing bacterial resistance and raises the risk of secondary infection. Recent studies have shown that even brief exposure to antibiotics can alter the composition of the intestinal microbiota for up to 2 years, and in cases of repeated exposure, the microbiota may never fully recover its original composition (Melander et al., 2018). Gerber et al. (2017) also note that, for acute respiratory infections, broad-spectrum agents do not offer any additional benefits over narrow-spectrum agents but are associated with significantly more adverse drug effects. Similarly, of the four first-line antitubercular drugs, three are narrow-spectrum—isoniazid, pyrazinamide, and ethambutol—which have little or no activity outside the *Mycobacterium* genus. However, in cases of MDR-TB, multiple broad-spectrum antibiotics are used for periods of up to 2 years (Melander et al., 2018). The use of compounds 5n and 5b in patients undergoing standard TB therapy could shorten treatment duration, reduce side effects, and limit the development of antibiotic resistance. These narrow-spectrum *Bfx* derivatives are particularly valuable for targeted treatment of both sensitive and resistant mycobacterial strains, reducing reliance on prolonged broad-spectrum antibiotics and minimizing microbiota disruption (Alm and Lahiri, 2020). The importance of our findings is supported by the claims of Collins and Riley (2022) who state that treatment regimens are now shifting toward the use of new narrow-spectrum antimicrobial agents. However, further clinical studies are needed to assess their safety and efficacy in combination with current therapies.

Cytotoxicity studies revealed that compound 5n has high selectivity and low toxicity, with an IC_50_ greater than 230.24 μM and 221.52 μM in the J774.A1 and MRC-5 cell lines, respectively, resulting in selectivity indices (SI) greater than 2,400. Although compound 5b showed higher cytotoxicity values (IC_50_ of 40.83 ± 10.72 μM for J774.A1 and 57.07 ± 48.15 μM for MRC-5), it also presented a high SI, highlighting the safety of these compounds for therapeutic use. Previous studies have established that compounds with an SI greater than 10 are highly selective, those between 1 and 10 indicate moderate selectivity, and values below 1 indicate low selectivity (Peña-Morán et al., 2016). Elisha et al. (2017) argue that an SI greater than 2.5 is acceptable, reinforcing the safety of these compounds, with extremely favorable SIs of 57 and 2,400 for 5b and 5n, respectively.

Synergism studies showed that *Bfx* derivatives can be effectively combined with RFP, presenting FICI values of 0.083 and 0.11 for compounds 5n and 5b, respectively. According to the criteria established by Caleffi-Ferracioli et al. (2013), FICI values less than 0.5 indicate synergy, values in the range of 0.5 to 4 are considered neutral, and values greater than 4 are considered antagonistic. The results indicate a synergistic interaction between both Bfx compounds and RIF *in vitro*, with compound 5n standing out particularly. These findings are highly significant, given that the recommended treatment for TB involves a combination of drugs with various mechanisms of action. The observed synergy can not only enhance the antimicrobial activity against *Mtb* but could also have significant clinical implications. Firstly, the synergistic effect could allow a reduction in the effective doses of each drug without compromising therapeutic efficacy. This dose reduction can minimize the adverse side effects often associated with higher doses of antitubercular medications, thereby improving patient compliance. Secondly, the enhanced bactericidal activity could lead to a shorter duration of therapy and could help overcome existing drug resistance.

Scanning electron microscopy revealed severe damage to the morphology of *Mtb* following treatment with *Bfx* 5n, showing distortions in bacillary structures, suggesting a potent bactericidal mechanism of action. Additionally, whole-genome sequencing of the 5n-resistant isolate identified resistance mechanisms mediated by the PPE family genes and *Rv1855c*. The PPE genes, associated with type VII secretion systems in *Mtb*, encode proteins that play a crucial role in membrane association, virulence factors, and immune evasion, although they are not essential for mycobacterial survival (Phelan et al., 2016; DeJesus et al., 2017). Flores-Valdez et al. (2009) report that the Rv1855c gene encodes a tetrahydromethanopterin reductase (THMR), an oxidoreductase enzyme dependent on the F420 coenzyme. This enzyme belongs to the luciferase-like monooxygenase family (PF00296) and plays a crucial role in various redox reactions within *Mtb* (Selengut and Haft, 2010). According to Purwantini et al. (2016), although THMR is commonly found in archaea, its mycobacterial homolog, F420-dependent glucose-6-phosphate dehydrogenase (G6PD420), encoded by the *Rv2951c* gene, is essential for the pathogen’s defense against oxidative and nitrosative stress (Davis et al., 2021). This is particularly relevant given that Bfx compounds release NO in biological media. F420-dependent enzymes, such as THMR (Rv1855c) and G6PD420 (Rv2951c), could be involved in the reduction of nitro groups in nitroaromatic compounds, facilitating the activation of these prodrugs. The enzymatic reduction of the nitro group leads to the release of NO, enhancing the antimicrobial effect of Bfx compounds (Chugunova et al., 2015). The interaction between this oxidoreductase and compound 5n suggests a possible synergy with other antitubercular drugs, such as pretomanid and delamanid, which are also activated by the F420 cofactor, produced by G6PD420 (Greening et al., 2016). We infer that 5n could potentiate the activity of these commercial drugs, improving their synergy and, consequently, the efficacy of the treatment.

Moreover, the MycoBrowser and PaxDb databases (MycoBrowser, 2024; PaxDb, 2024) highlight functional similarities between *Rv1855c* and *Rv2951c*. The *Rv2951c* gene is involved in the modification of the lipid fraction of phenolic trehalose dimycolates (PDIM) and the glycosidic domain of phenolic glycolipid lipids (PGL) (Jackson et al., 2021). These lipids are part of the cell wall, and their possible inhibition could cause structural damage, as observed in microscopy results, which could lead to the death of the mycobacterium. Furthermore, These lipids contribute to increasing its virulence, as well as reducing the host’s immune response, cytokine production, and T cell signaling (Sinsimer et al., 2008; Rahlwes et al., 2023). Given the similarities between these genes, it is likely that they are involved in the same biological processes. Furthermore, Tudó et al. (2010)) report that the expression of the *Rv1855c* gene increases when *Mtb* is treated with isoniazid, suggesting its involvement in resistance mechanisms. For all these reasons, Rv1855c emerges as a promising target to combat *Mtb*, especially in resistant strains.

*In silico* studies showed that compound 5n strongly binds to the protein expressed by the *Rv1855c* gene, with electrostatic, hydrogen, and hydrophobic interactions resulting in an affinity of up to −8.2 kcal/mol. These results are close to those obtained by Rossetti et al. (2020), who reported an affinity of −8.6 kcal/mol when docking RPF with its rpoB receptor. Additionally, According to Coumar (2021), high affinity is characterized by a binding energy of −7 kcal/mol or lower, indicating a strong and stable interaction. However, Given that Rv1855c is a hypothetical protein with limited information regarding its possible functions in Mtb, we cannot assert that the mechanism of action by which our compound acts against Mtb is through the inhibition of this protein. Nevertheless, based on its homologous protein, we cannot entirely rule out this possibility either. Therefore, further studies are recommended to validate the role of this protein in resistance mechanisms. Finally, considering that *Mtb* acts as a facultative intracellular pathogen in humans, it was crucial to assess the ability of these compounds to target the interior of macrophages and inhibit intracellular mycobacteria (Protopopova et al., 2005; Kornfeld et al., 1999). Disrupting *Mtb*’s survival strategies within macrophages could reduce treatment duration and the required antibiotic concentrations (Niller et al., 2017; Campos et al., 2022).

The Assays demonstrated that Bfx derivatives exhibit superior intracellular activity compared to RFP. Furthermore, consistent with the findings reported by Matsumoto et al. (2006), compound 5n displays greater intramacrophagic activity than delamanid and pretomanid, as evidenced by higher inhibition at a concentration of 0.09 μM. In contrast, delamanid and pretomanid achieved inhibitions of 0.7 and 0.2 log₁₀ CFU/mL, respectively, at a concentration of 1 μg/mL. Although it is acknowledged that the experimental conditions were not identical, these results provide an approximation of the potential of compound 5n compared to commercial drugs. However, additional studies under standardized experimental conditions are necessary to confirm and further elucidate these findings. The superiority of compound 5n over RFP and 5b in reducing pulmonary bacterial load in the *in vivo* tests highlights its potential as a more effective therapeutic option against Mtb. These results not only reflect 5n’s ability to penetrate and act in hostile intracellular environments, such as acidified phagosomes, but also underscore the importance of its chemical structure, particularly the presence of the pyridine ring, which may contribute to its enhanced antimicrobial activity (Villamizar-Mogotocoro et al., 2020).

In conclusion, *Bfx* derivatives, particularly compound 5n, exhibit substantial therapeutic potential against MDR-*Mtb* strains. Their potent *in vivo* efficacy, favorable toxicity profile, and observed synergy with RFP support further optimization and preclinical evaluation. This approach is consistent with recommended criteria for anti-tuberculosis lead compounds (Fernandes et al., 2022). Additional mechanistic studies are warranted to elucidate the precise mode of action of *Bfx* 5n, which could provide valuable insights for the development of novel anti-tuberculosis agents.

## Data Availability

The datasets presented in this study can be found in online repositories. The names of the repository/repositories and accession number(s) can be found in the article/Supplementary material.

## Footnotes

^1^https://zhanggroup.org/I-TASSER/

^2^https://pockdrug.rpbs.univ-paris-diderot.fr/cgi-bin/index.py?page=Home
