# Supplementary material for: Screening of novel narrow-spectrum benzofuroxan derivatives for the treatment of multidrug-resistant tuberculosis through in silico, in vitro, and in vivo approaches
Source: Front Microbiol. 2024 Oct 11;15:1487829. doi: 10.3389/fmicb.2024.1487829 (PMC11502347; doi:10.3389/fmicb.2024.1487829)
Supplement: Supplementary file 1 [file Data_Sheet_1.PDF]

# Screening of Novel Narrow-Spectrum Benzofuroxan Derivatives for the Treatment of Multidrug-Resistant Tuberculosis through *In silico*, *In vitro*, and *In vivo* approaches

Débora Leite Campos[1†], Christian Shleider Carnero Canales[1,2†], Fernanda Manaia Demarqui[1], Guilherme F. S. Fernandes[3,4], Camila Gonçalves dos Santos[1], João Lucas B. Prates[3], Ingrid Gracielle Martins da Silva[5], Karine Brenda Barros-Cordeiro[5], Sônia Nair Báo[5], Leonardo Neves de Andrade[6], Nathália Abichabki[6], Luísa Vieira Zacharias[6], Marli Matiko Anraku de Campos[7], Jean Leandro dos Santos[3], Fernando Rogério Pavan[1*].

[1]Tuberculosis Research Laboratory, School of Pharmaceutical Sciences, São Paulo State University – UNESP, Araraquara, São Paulo, Brazil

[2]School of Pharmacy, biochemistry and biotechnology, Santa Maria Catholic University, Arequipa, Perú

[3]Medicinal Chemistry Laboratory, School of Pharmaceutical Sciences, São Paulo State University – UNESP, Araraquara, São Paulo, Brazil

[4]School of Pharmacy, University College London, 29-39 Brunswick Square, London, United Kingdom

[5]Microscopy and Microanalysis Laboratory, Cell Biology Department, Institute of Biological Sciences, University of Brasilia, Brasília, Brazil

[6]School of Pharmaceutical Sciences of Ribeirão Preto, University of São Paulo – USP, Ribeirão Preto, São Paulo, Brazil.

[7] Mycobacteriology Laboratory, Department of Clinical and Toxicological Analysis, Federal University of Santa Maria, Santa Maria, Brazil

*Correspondence: fernando.pavan@unesp.br

†These authors contributed equally to this work

**Materials and methods**

Sabouraud, Mueller-Hinton II, Middlebrook 7H9, and 7H11 culture media were obtained from Kasvi (Paraná, Brazil). Catalase was purchased from Thermo Fisher Scientific Inc. (MA, USA). Bovine albumin was obtained from Interlab Confiança (São Paulo, Brazil). Modified Eagle Medium (DMEM) and Roswell Park Memorial Institute medium 1640 (RPMI) were acquired from Gibco®, fetal bovine serum (FBS), dextrose, gentamicin sulfate, amphotericin B, isoniazid (INH), RFP, linezolid (LNZ), amikacin (AMK), gatifloxacin (GAT)/moxifloxacin (MOX), and amikacin (AMK) were acquired from Sigma-Aldrich®.

**Strains and Bacterial Culture**

Macrophage cells (J774A.1 ATCC TIB-67) were cultured in RPMI medium supplemented with 10% fetal bovine serum (FBS), gentamicin sulfate (50 mg/L), and amphotericin B (2 mg/L) under incubation at 37°C with 5% $CO_2$. Fibroblasts cells (MRC-5) were cultured in DMEM medium supplemented with 10% fetal bovine serum (FBS), gentamicin sulfate (50 mg/L), and amphotericin B (2 mg/L) under incubation at 37°C with 5% $CO_2$. Mtb H37Rv (ATCC 27294) was used for most of the experiments described. The strain was cultured in Middlebrook 7H9 broth supplemented with 0.5% glycerol and 10% OADC (oleic acid, albumin, dextrose, catalase) under agitation at 37°C. Clinical isolates of Mtb, *Mycobacterium smegmatis mc²155* (ATCC 700084), and *Mycobacterium bovis* TMC1011 (ATCC 35734) were cultured under the same conditions. Gram-positive and Gram-negative bacteria were cultured in Mueller-Hinton II medium (Ben Hur et al., 2022), and *Candida albicans* was cultured on Sabouraud agar.

**Benzofuroxan Compounds**

Stock solutions were prepared at a concentration of 10 mg/mL in DMSO for *in vitro* experiments. For *in vivo* treatment, suspensions were prepared at a concentration of 200 mg/mL in sunflower oil, which were sonicated for 30 minutes one hour before treatment.

**Antimicrobial Evaluation against Gram-positive, Gram-negative Bacteria, and *Candida albicans***

For *Candida albicans*, the methodology of Rodríguez-Tudela et al. (Rodríguez-Tudela et al., 2001) was employed. Briefly, cultures were performed on Sabouraud agar, and after 48 hours of incubation, colonies were collected to prepare the inoculum in broth. Twenty mL aliquots of Sabouraud dextrose broth were placed in 50 mL Falcon tubes, and yeast scrapings from the initial plate were added. The solution was incubated for 48 hours at 37°C without agitation until use. The concentration was evaluated by fluorescence at 530 nm, adjusting it to absorbance values between 0.1 and 0.15, corresponding to $1 \times 10^6$ CFU/mL, and the inoculum was diluted to $2 \times 10^3$ CFU/mL for use. In a 96-well microplate, Sabouraud broth with the compounds was distributed in serial dilutions ranging from 0.39 to 100 µg/mL. After 48 hours of incubation under the same conditions, absorbance at 570 nm was read, followed by resazurin application, and fluorescence was measured at 530/590 nm.

**Results**

**Table S1.** Evaluation of the synergistic effect of Bfx and RFP using the checkerboard assay

| Strain | Compounds | $MIC_{90}$ (µM) | $MIC_{90}$ combined(µM) | FICI | Results |
|---|---|---|---|---|---|
| *Mtb H37Rv* | **5n** | 0,09 | 0,00578 | 0,083 | Synergistic |
| | RFP | 0,05 | 0,00098 | | |
| | **5b** | 0,7 | 0,0625 | 0.11 | Synergistic |
| | RFP | 0,05 | 0,00098 | | |

**Table S2.** Detailed Information on the Druggability Pockets of the Rv1855c protein

| Pocket | Vol. Hull | H. Kyte | P. Res. | A. Res. | O. Atom | Nb. Res. | Drug Prob | S. Deviation |
|---|---|---|---|---|---|---|---|---|
| 12 | 434.93 | 1.35 | 0.29 | 0.14 | 0 | 14 | 0.99 | 0 |
| 9 | 325.74 | 1.17 | 0.44 | 0.11 | 0.04 | 9 | 0.98 | 0.01 |
| 15 | 537.06 | 1 | 0.33 | 0 | 0 | 9 | 0.95 | 0.01 |
| 0 | 3811.55 | 0.28 | 0.32 | 0.16 | 0 | 37 | 0.94 | 0.02 |
| 6 | 276.69 | 0.76 | 0.36 | 0 | 0 | 11 | 0.9 | 0.03 |
| 13 | 434.13 | -0.26 | 0.6 | 0.3 | 0.03 | 10 | 0.85 | 0.02 |
| 7 | 318.54 | 0.3 | 0.5 | 0 | 0 | 8 | 0.78 | 0.06 |
| 3 | 498.79 | -0.19 | 0.47 | 0.13 | 0 | 15 | 0.68 | 0.02 |
| 1 | 1161.3 | 0.02 | 0.53 | 0 | 0 | 17 | 0.67 | 0.07 |
| 5 | 404.23 | -1.09 | 0.67 | 0.42 | 0 | 12 | 0.48 | 0.2 |
| 2 | 364.61 | -0.55 | 0.45 | 0.09 | 0 | 11 | 0.39 | 0.03 |
| 16 | 262.3 | -1.04 | 0.43 | 0.29 | 0 | 7 | 0.35 | 0.11 |
| 11 | 379.47 | -0.99 | 0.45 | 0.09 | 0 | 11 | 0.18 | 0.02 |
| 8 | 363.01 | -1.5 | 0.7 | 0.1 | 0 | 10 | 0.06 | 0.01 |
| 10 | 421.1 | -2.06 | 0.75 | 0.08 | 0 | 12 | 0.02 | 0 |
| 4 | 253.68 | -2.16 | 0.71 | 0.14 | 0 | 7 | 0.02 | 0 |
| 14 | 224.72 | -2.3 | 0.67 | 0 | 0 | 6 | 0.01 | 0 |

*Vol. Hull = Volume Hull; H. Kyte= Hydrophobic Kyte; P. Res.= Polar Residues Proportion; A. Res.= Aromatic Residues Proportion (F,Y,H,W); O. Atom = Otyr atom; Drug Prob= Druggability Probability; Nb. Res.= Number of pocket residues

**Table S3.** Comparative analysis of docking performance across druggable pockets

| Pocket | Mode | Affinity (kcal/mol) | RMSD L.B. | RMSD U.B. |
|---|---|---|---|---|
| 9 | 1 | -7.6 | 0 | 0 |
| | 2 | -7.2 | 27.779 | 30.903 |
| | 3 | -6.8 | 29.86 | 32.919 |
| | 4 | -6.7 | 3.964 | 12.674 |
| | 5 | -6.7 | 3.594 | 6.237 |
| | 6 | -6.6 | 5.905 | 11.031 |
| | 7 | -6.5 | 11.481 | 16.619 |
| | 8 | -6.3 | 9.665 | 12.886 |
| | 9 | -6.3 | 4.343 | 12.2 |
| 12 | 1 | -8.1 | 0 | 0 |
| | 2 | -7.7 | 2.534 | 3.347 |
| | 3 | -7.5 | 6.391 | 7.464 |
| | 4 | -7.2 | 6.322 | 9.98 |
| | 5 | -7.2 | 1.569 | 2.252 |
| | 6 | -7 | 6.54 | 14.411 |
| | 7 | -6.9 | 3.425 | 5.868 |
| | 8 | -6.9 | 4.201 | 12.179 |
| | 9 | -6.8 | 7.655 | 8.537 |
| 15 | 1 | -8.2 | 0 | 0 |
| | 2 | -7.8 | 4.897 | 12.66 |
| | 3 | -7.6 | 5.19 | 12.952 |
| | 4 | -7.5 | 4.073 | 12.222 |
| | 5 | -7.4 | 6.789 | 7.914 |
| | 6 | -7.4 | 4.543 | 12.451 |
| | 7 | -7 | 7.711 | 13.034 |
| | 8 | -6.8 | 7.348 | 8.285 |
| | 9 | -6.8 | 5.118 | 12.625 |

*RMSD L.B.= Root Mean Square Deviation Lower Bound; RMSD U.B.= Root Mean Square Deviation Upper Bound

**Table S4.** types of binding interactions, distances, and categories of Bfx with druggable pockets of the Rv1855c protein

| Pocket | Interaction | Distance | Category | Types |
|---|---|---|---|---|

| | | | | |
|---|---|---|---|---|
| 9 | :UNK1:O - A:TYR87:O | 3.326 | Hydrogen Bond | Conventional Hydrogen Bond |
| | :UNK1 - A:ARG88 | 4.3788 | Hydrophobic | Pi-Alkyl |
| | :UNK1 - A:LEU92 | 5.45178 | Hydrophobic | Pi-Alkyl |
| | :UNK1 - A:VAL18 | 5.21005 | Hydrophobic | Pi-Alkyl |
| | :UNK1 - A:PRO58 | 4.36529 | Hydrophobic | Pi-Alkyl |
| | :UNK1 - A:LEU60 | 5.37038 | Hydrophobic | Pi-Alkyl |
| | :UNK1 - A:ARG88 | 3.73084 | Hydrophobic | Pi-Alkyl |
| 12 | A:GLY184:HN - :UNK1:O | 2.59085 | Hydrogen Bond | Conventional Hydrogen Bond |
| | :UNK1:C - A:GLN8:OE1 | 3.52464 | Hydrogen Bond | Carbon Hydrogen Bond |
| | :UNK1 - A:TRP116 | 4.55648 | Hydrophobic | Pi-Pi T-shaped |
| | :UNK1 - A:TRP116 | 5.4073 | Hydrophobic | Pi-Pi T-shaped |
| | A:TRP116 - :UNK1 | 5.01846 | Hydrophobic | Pi-Pi T-shaped |
| | :UNK1 - A:LEU234 | 5.27918 | Hydrophobic | Alkyl |
| | :UNK1 - A:ALA285 | 4.65024 | Hydrophobic | Pi-Alkyl |
| | :UNK1 - A:VAL82 | 5.37934 | Hydrophobic | Pi-Alkyl |
| 15 | A:GLY184:HN - :UNK1:O | 2.52025 | Hydrogen Bond | Conventional Hydrogen Bond |
| | A:GLY182:HN - :UNK1 | 3.19934 | Hydrogen Bond | Pi-Donor Hydrogen Bond |
| | :UNK1 - A:TRP116 | 4.57986 | Hydrophobic | Pi-Pi T-shaped |
| | :UNK1 - A:TRP116 | 5.33698 | Hydrophobic | Pi-Pi T-shaped |
| | A:TRP116 - :UNK1 | 4.9635 | Hydrophobic | Pi-Pi T-shaped |
| | :UNK1 - A:LEU234 | 5.11611 | Hydrophobic | Alkyl |
| | :UNK1 - A:ALA285 | 4.85598 | Hydrophobic | Pi-Alkyl |
| | :UNK1 - A:VAL82 | 5.47661 | Hydrophobic | Pi-Alkyl |

*UNK1= **5n**

## Reference


Ben Hur, D., Kapach, G., Wani, N. A., Kiper, E., Ashkenazi, M., Smollan, G., Keller, N., Efrati, O., & Shai, Y. (2022). Antimicrobial Peptides against Multidrug-Resistant Pseudomonas aeruginosa Biofilm from Cystic Fibrosis Patients. Journal of Medicinal Chemistry, 65(13), 9050–9062. https://doi.org/10.1021/acs.jmedchem.2c00270

Rodríguez-Tudela, J. L., Cuenca-Estrella, M., Díaz-Guerra, T. M., & Mellado, E. (2001). Standardization of Antifungal Susceptibility Variables for a Semiautomated Methodology. Journal of Clinical Microbiology, 39(7), 2513–2517. https://doi.org/10.1128/JCM.39.7.2513-2517.2001
